# Myocardial Late Enhancement With Photon-Counting Detector CT in Spontaneous Coronary Artery Dissection: Prospective Comparison With Cardiac MRI

**DOI:** 10.1097/RLI.0000000000001203

**Published:** 2025-04-25

**Authors:** Konstantin Klambauer, Ernst Klotz, Lukas J. Moser, Tobias Kälin, Andrea Biondo, Victor Schweiger, Victor Mergen, Costanza Lisi, Michael Würdinger, Rabea Schlenker, Davide Di Vece, Alexander Gotschy, Martin Reiner, Jelena-R. Ghadri, Verena C. Wilzeck, Matthias Eberhard, Christian Templin, Robert Manka, Hatem Alkadhi

**Affiliations:** Diagnostic and Interventional Radiology, University Hospital Zurich, University of Zurich, Zurich, Switzerland (K.K., L.J.M., T.K., A.B., V.M., C.L., R.S., A.G., M.R., V.C.W., M.E., R.M., H.A.); Siemens Healthineers AG, Forchheim, Germany (E.K.); Department of Cardiology, University Heart Centre, University Hospital Zurich, Zurich, Switzerland (V.S., M.W., R.S., A.G., M.R., J.-R.G., V.C.W., R.M.); Department of Biomedical Sciences, Humanitas University, Milan, Italy (C.L.); Department of Cardiology and Internal Medicine B, University Medicine Greifswald, Greifswald, Germany (D.D.V., C.T.); Institute for Biomedical Engineering, University and ETH Zurich, Zurich, Switzerland (A.G., V.C.W.); Center for Molecular Cardiology, Schlieren Campus, University of Zurich, Zurich, Switzerland (C.T.); and Swiss CardioVascularClinic, Private Hospital Bethanien, Zurich, Switzerland (C.T.)

**Keywords:** spontaneous coronary artery dissection, photon-counting detector CT, late enhancement imaging, cardiac MR imaging, myocardial tissue characterization

## Abstract

**Objectives:**

Spontaneous coronary artery dissection (SCAD) is a rare cause of acute coronary syndrome and myocardial infarction. Accurate diagnosis is crucial for appropriate management. This study aimed to compare late enhancement (LE) imaging using photon-counting detector (PCD)-CT with cardiac MRI in patients with SCAD in the acute phase and during follow-up and to introduce a novel approach for visualizing myocardial extracellular volume (ECV) distribution in the myocardium.

**Materials and Methods:**

This single-center prospective study enrolled patients with SCAD diagnosed with invasive coronary angiography. LE iodine imaging with spectral dual-source PCD-CT and cardiac MRI was performed early after symptom onset and at short-term follow-up. CT included coronary angiography and LE imaging (5 minutes after contrast). LE CT was assessed using the combination of conventional LE images, overlay images, polar maps, and with newly developed atlas maps. Atlas maps represent 2-dimensional maps with prefiltering applied to enable a simpler and more intuitive reading of ECV distribution across the myocardium. Cardiac MRI served as the reference standard for identifying pathologic myocardial segments based on late gadolinium enhancement (LGE) and edema on T2-weighted and T2-mapping images. Agreement between modalities was evaluated using Cohen's κ.

**Results:**

Seventeen patients (median age, 44 years [interquartile range, 36–52]; 11 women) underwent 24 LE CT and cardiac MRI scans. Sixteen patients (median age, 44 years; 10 women) underwent acute phase imaging (median 6 days after symptom onset), and 8 patients (median age, 45 years; 6 women) underwent follow-up imaging (median 120 days after symptom onset). Atlas maps were helpful in detecting segments with pathological ECV and to adjudicate corresponding myocardial segments. Agreement between LE CT with LGE cardiac MRI was strong in the acute phase (κ = 0.832), improving to almost perfect when comparing LE-CT with both LGE and edema in cardiac MRI (κ = 0.944). At follow-up imaging, agreement further improved as edema resolved (κ = 0.956).

**Conclusions:**

LE imaging with PCD-CT demonstrated strong agreement with cardiac MRI for detecting myocardial injury in SCAD, which further improved at follow-up when edema resolved. Newly introduced atlas maps proved useful for a simple and intuitive visualization of myocardial injury.

Spontaneous coronary artery dissection (SCAD) represents a rare form of acute coronary syndrome predominantly affecting younger women. SCAD is linked to exogenous hormones, genetic arteriopathies, connective tissue disorders, and both physical and emotional stressors.^1^ Patients with SCAD typically present with acute chest pain, elevated cardiac biomarkers, and electrocardiogram (ECG) abnormalities indicative of acute myocardial infarction.^2^

Invasive coronary angiography (ICA) represents the gold standard for diagnosing SCAD,^1,3^ with the Yip-Saw classification characterizing different angiographic phenotypes.^4^ Cardiac magnetic resonance imaging (MRI) showing late gadolinium enhancement (LGE) and/or edema in a territory corresponding to a suspected dissection can help to confirm SCAD or suggest alternate diagnoses such as myocarditis.^5–7^

The role of cardiac computed tomography (CT) in patients with SCAD remains to be determined. While coronary CT angiography appears to be effective for detecting SCAD in larger coronary arteries, the diagnosis of SCAD in smaller, distal vessels remains challenging.^8–10^ Another option in cardiac CT is late iodine enhancement (LE) imaging, leveraging the same pathophysiological principles as LGE in cardiac MRI.^11–14^ Recent studies indicated that adding LE imaging to a coronary CT angiography protocol is beneficial in patients presenting with acute chest pain through the identification of causes other than coronary artery disease such as myocarditis and takotsubo cardiomyopathy, hereby improving the diagnostic yield of the examination.^15,16^

The recent advent of photon-counting detector (PCD)-CT has sparked further interest in myocardial characterization with CT.^17^ Compared with conventional energy-integrating detector CT, dual-source PCD-CT offers a lower noise, a higher signal- and contrast-to-noise ratio, and enables spectral data acquisition at high temporal resolution,^18–23^ which has proven beneficial for LE imaging.^24–26^

The aim of this study was to compare LE imaging using PCD-CT with cardiac MRI in patients with SCAD in the acute phase and during follow-up and to introduce a novel approach for visualizing myocardial extracellular volume (ECV) distribution in the myocardium.

## METHODS

### Study Population

This study complied with the Declaration of Helsinki guidelines and received ethics committee approval. All patients provided written informed consent and were enrolled in the SCAD registry at our hospital. Conducted as a single-center prospective trial, this study involved consecutive patients diagnosed with acute SCAD through ICA. Participant enrolment in this study started in March 2021 following the installation of a clinical PCD-CT scanner in our department and terminated in April 2024. As part of the SCAD registry research protocol, patients were planned to undergo cardiac CT and cardiac MRI in the acute phase during hospitalization and during follow-up at 2 months. Exclusion criteria were (a) less than 18 years of age, (b) pregnancy, (c) insufficient image quality of LE CT, and (d) corrupted raw data. Participant demographics were retrieved from electronic medical records.

### Cardiac MRI Scan Protocol and Image Assessment

Cardiac MRI was conducted on our clinical 1.5T scanners (Achieva; Philips, Amsterdam, the Netherlands or MAGNETOM Sola; Siemens Healthineers AG, Forchheim, Germany), both equipped with cardiac receiver arrays. ECG synchronization was used. Cardiac functional assessment included breath-hold balanced steady-state free precession (bSSFP) short-axis cine images covering the entire left ventricle (LV) and bSSFP long-axis cine images in 2, 3, and 4 chamber orientations. The protocol included T1-weighted and T2-weighted turbo-spin-echo black-blood sequences (with and without fat saturation) in short-axis orientation, T2 mapping, and inversion recovery LGE images acquired 10 minutes after administering 0.2 mmol/kg gadolinium-based contrast media (Gadovist; Bayer Pharma AG, Berlin, Germany). The inversion recovery prepulse delay was determined using a Look-Locker sequence.

Two readers ([A.B.] and [R.M.], - with 4 and 20 years of experience in cardiac MRI, respectively) assessed MRI scans with dedicated software (Intellispace Portal. Version 9.0, Philips, Amsterdam, the Netherlands). In case of disagreement, a final diagnosis was reached by the more experienced reader. Readers were blinded to patient identities and to the results from CT. Myocardial segmentation was performed using the 16-segment American Heart Association (AHA) model. Hyperenhanced segments on LGE MRI were documented for both the subendocardium and subepicardium (total of 2*16 = 32 segments per scan). The presence of microvascular obstruction (MVO) was recorded when hypoenhanced areas were observed within the hyperenhanced infarct regions. Edema assessment was performed for the entire myocardial wall using T2-weighted and T2-mapping images (total of 16 segments per scan). Segments were classified as edematous if T2 values exceeded 2 standard deviations above scanner reference values, automatically determined by the scanner software, with motion correction applied.

### CT Scan Protocol

Scans were performed using a first-generation dual-source PCD-CT system (NAEOTOM Alpha; Siemens Healthineers AG, Forchheim, Germany) equipped with two photon-counting detectors (cadmium telluride) with a 144 × 0.4-mm collimation and a gantry rotation time of 0.25 seconds. First, a nonenhanced scan for calcium scoring was performed with an image quality level of 20 at 75% of the RR interval. Then, 60–80 mL of intravenous iodinated contrast medium (iopromidum 370 mg I/mL, Ultravist; Bayer Pharma AG, Berlin, Germany) was injected, followed by a 20 mL saline chaser (NaCl 0.9%) at a flow rate of 5 to 6 mL/s, depending on the weight of the patient. Coronary CT angiography scans were acquired in the ECG-triggered sequential spectral (QuantumPlus) mode with a tube voltage of 120 kVp and variable ECG pulsing depending on the average and variability of heart rate. LE scans were acquired 5 minutes after start of the contrast media injection using an ECG-triggered prospective systolic triggering at 280 ms of the RR interval, as previously recommended,^24,25^ and at an image quality level of 80. This scan mode is robust against arrhythmia and ectopic beats. The median heart rate during LE CT data acquisition was 66/min (interquartile range [IQR], 60–75 /min).

### CT Image Reconstruction and Extracellular Volume Analysis

Cardiac LE scans were reconstructed as virtual monoenergetic images (VMI) at 65 keV with 1.5-mm slice thickness and 1-mm increment using a quantitative soft tissue kernel (Qr40) and applying quantum iterative reconstruction level 3.^24,25^ Coronary CT angiography scans were reconstructed with identical settings. Iodine images were derived from spectral cardiac LE scans, and ECV was calculated using prototype software (CT Cardiac Functional Analysis 3.0; Siemens Healthineers AG, Forchheim, Germany) through spectrally derived iodine concentrations in the myocardium and blood pool. The hematocrit (L/L), determined from the patient's blood sample within 24 hours of the scan, was incorporated into the calculation using the following formula:


$$\text{ECV}=\left(1-\text{hematocrit)}\times {\text{[Iodine}}_{\text{cardium}}\right]/\left[{\text{Iodine}}_{\text{blood}}\right]$$

The software enables transmural ECV calculations by restricting analysis to specific radial profile ranges (subendocardium: inner 10% to 50% and subepicardium: 50% to outer 90% of the myocardial wall thickness).

### Overlay Images and Polar Maps

Three-dimensional analysis was performed by generating a LV heart model from an end-systolic coronary CT angiography image dataset at 65 keV, or from an end-systolic LE image dataset when coronary CT angiography images were not available at end-systole. ECV volume data derived from LE VMI and iodine images were aligned with the LV model using nonrigid registration and overlaid on the LV model. The ECV data were presented and exported as 16-segment polar maps being color coded to indicate ECV levels for the subendocardium and subepicardium.

### Atlas Maps

In addition, atlas maps were developed in this study with the aim to enable a simpler and more intuitive reading of ECV distribution across the myocardium than using polar maps. The primary challenge of LE CT imaging is the inherently low iodine concentration during the quasi-equilibrium phase, especially when using routine doses (22–30 g) as in this study. Compared to the 0.2 mmol/kg of gadolinium contrast media used for LGE MRI, the resulting signal from CT is therefore lower. Conversely, CT offers full 3-dimensional analysis of the whole heart at substantially higher spatial sampling: the typical voxel size in this study was 0.4 × 0.4 × 1.0 mm; there are usually well over 500,000 voxels available for analysis. While PCD-CT is practically free of electronic noise, contributions of the inherent random statistical fluctuations of measuring photons are unavoidable and lead to a relatively low signal-to-noise ratio in iodine images at this high level of resolution. As this noise is completely random, however, it can be substantially reduced by averaging over enough voxels. Recent work has shown that by averaging over suitable anatomical sub compartments, for example, 16 myocardial AHA (American Heart Association) segments split into subendo- and subepicardium layers, the precision is high enough to reliably measure regional variations down to one % ECV.^13^

For the detection of focal lesions, we use this concept but with sampling compartments large enough to reduce noise but still small enough not to blur lesions. The compartments are constructed from the automatic heart model with prototype software (Cardiac Functional Analysis Prototype, Siemens) by subdividing the heart along the long axis into 34 short-axis layers (3 in AHA 16) and into 72 angular segments (6 or 4 in AHA 16). Voxels are collected by a raytracing algorithm originating from the long axis that allows to separately collect data for the subendocardium, the midwall, and the subepicardium. This data is presented per layer with a continuous color lookup table in the form of “AHA similar” polar maps (see supplementary figure, http://links.lww.com/RLI/B32). Additionally, the mean ECV values and the corresponding number of voxels for each of the 3 × 34 × 72 segments are exported in platform independent “csv” format. The choice of 34 × 72 was motivated by practical considerations and the aim to roughly adjust the segment size to the size of a typical LGE MRI voxel. For a heart of 7-cm height and 5-cm diameter, each segment spans about 2 × 2 mm on the surface and with a wall thickness of 1 cm roughly covers a volume of 20 mm^3^.

In PCD-CT, LE imaging and ECV determination are practically equivalent. Therefore, we speculated that it might be possible to classify and automatically detect focal lesions solely based on their ECV value. To do so, we interpreted each of the 3 circular layers as a 34 × 72 matrix arbitrarily cut open anteriorly (center of segment AHA 1), which was further processed to remove remaining non connected speckle noise still present in the polar maps. This was achieved using 5 by 5 median filtration. A filter size of 5 achieves a noticeable noise reduction but is still small enough that lesions of about 1-cm diameter on the surface are fully retained. Simulations showed that 5 by 5 median filtering reduced Gaussian noise by about a factor of four. In comparison, a 3 by 3 median filtering, which we had also considered, only achieved a little more than a factor of 2 and was therefore considered less suited for subsequent thresholding. The filtered data were then subjected to thresholding using 45%, 40%, and 35% ECV as markers of a descending probability for the presence of a focal lesion. The thresholds were selected from first principles (values below 30% are most likely normal, values above 50% almost certainly positive) and from a pilot histogram analysis of a few patient data not included in this study.

The results were then graphically coded (see supplementary figure, http://links.lww.com/RLI/B32) for the 3 layers with the addition of the volume percentage of the respective layer with ECV values above 45%, 40%, and 35%. These volume percentages help to better assess the degree of transmurality of a lesion. They can also be used to absolutely quantify scar size. The filtering and atlas generation was done with offline in-house code programmed in R (R Core Team, Vienna, Austria), the results were generated as “pdf” files.

### CT Image Assessment and Comparison With Cardiac MRI

Two independent radiologists ([K.K.] and [H.A.] with 6 and 20 years of experience in cardiac CT, respectively) assessed CT images, with (K.K.) conducting the assessment twice after a time interval of 4 weeks to avoid recall bias. Both readers were blinded to patient identity and to the results from cardiac MRI. For each patient, LE images, overlay images in short-axis and 2-chamber long-axis views, as well as polar and atlas maps were evaluated. A myocardial segment was deemed pathologic if one of the following criteria was met:

- LE images showing hyperenhancement;
- Areas with ECV elevation >45% in overlay images;
- >50% of the segment showing ECV elevation >45% in polar maps;
- >25% of the segment showing ECV elevation >40% in atlas maps.

This process was performed for the subendocardium and subepicardium of each scan (total of 2*16 = 32 segments).

These thresholds were selected conservatively to minimize false-positive identification of myocardial injury in the absence of a validated standard for SCAD-related ECV elevation. The cutoffs were not derived from the study dataset but rather adapted from published literature on ischemic and nonischemic cardiomyopathies^11,19^ while accounting for the technical properties of polar and atlas maps. A higher threshold (45%) was used for polar maps to ensure specificity, while a slightly lower threshold (40%) was used for atlas maps due to their additional filtering and smoothing effects.

Intermodality concordance between CT and cardiac MRI was defined as follows:

- Total concordance: CT identified the same pathologic segments as cardiac MRI.
- Partial concordance: CT identified more or less pathologic segments than cardiac MRI, but with overlap between modalities.
- No concordance: CT identified none of the pathologic segments according to cardiac MRI.

The signal-to-noise ratio was calculated as the mean iodine value of the aortic lumen in the LE phase divided by its standard deviation.

### Statistical Analyses

Categorical variables were reported as counts and percentages, while continuous variables were presented as medians with interquartile ranges (IQR) for nonnormally distributed data (Shapiro-Wilk test). Paired comparisons between acute and follow-up imaging were performed using the Wilcoxon signed-rank test. Cohen's weighted κ statistic was used to assess interreader and intermodality agreement because mixed-effects logistic regression did not indicate strong dependence among segments within patients (random effect variance = 0). Agreement was interpreted as follows: <0.20, no agreement; 0.20–0.39, minimal; 0.40–0.59, weak; 0.60–0.79, moderate; 0.80–0.89, strong; 0.89–1.00, almost perfect.^27^ Intermodality agreement was evaluated for LE CT with LGE cardiac MRI and with LGE plus edema on cardiac MRI. A segment was classified as affected if either the subendocardium or subepicardium was involved, ensuring comparability with edema readouts that did not differentiate between subendocardial and subepicardial involvement. Analyses were performed using R version 4.4.0 (R Core Team, Vienna, Austria).

## RESULTS

### Study Population

**FIGURE 1:**
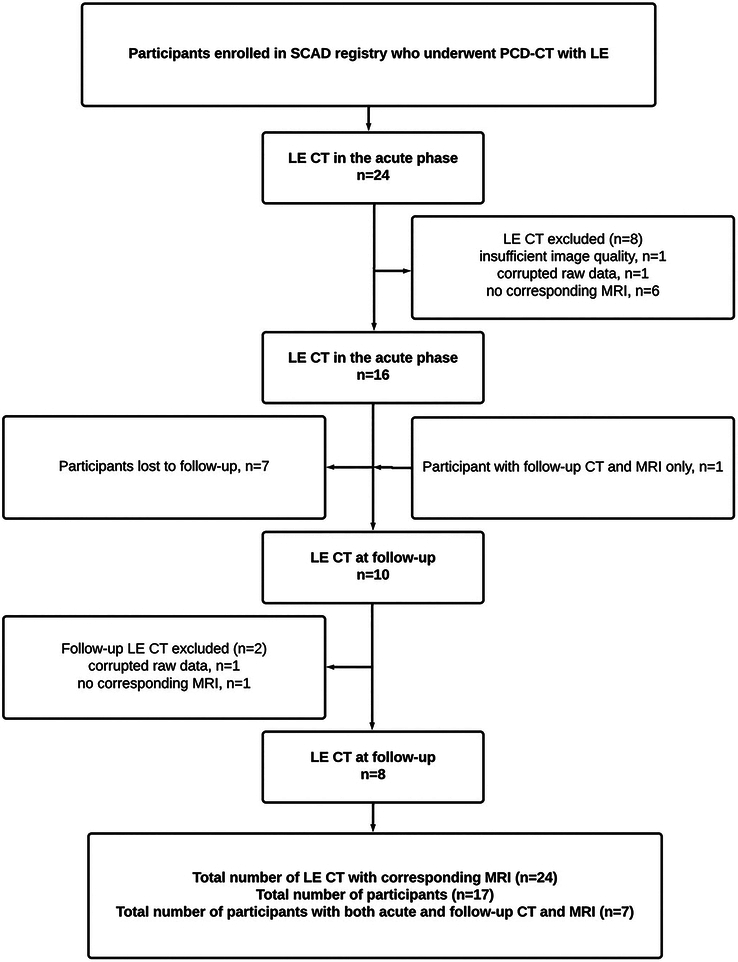
Study flowchart of the study. LE, late iodine enhancement; PCD, photon-counting detector; SCAD, spontaneous coronary artery dissection.

Twenty-four patients were enrolled. During the acute phase, 8 scans were excluded because of insufficient image quality caused by artifacts from an extracorporeal foreign body (n = 1), corrupted raw data (n = 1), and absence of cardiac MRI (n = 6). Seven of the 16 patients were lost to follow-up. One patient not included in the acute phase underwent follow-up imaging with both CT and MRI. Of the 10 patients who underwent follow-up imaging, 2 were excluded because of artifacts from an extracorporeal foreign body (n = 1) and missing corresponding cardiac MRI (n = 1). Thus, the final analysis included 17 patients with a total of 24 CT and MRI scans (Fig. 1). None of the included patients had a known history of coronary artery disease, prior myocardial infarction, or ischemic or nonischemic cardiomyopathy.

The study population included 6 males (6/17, 35%) and 11 females (11/17, 65%), with a median age of 44 years (IQR, 36–52 years) (Table 1). The left anterior descending artery was the most frequently affected artery (8/17, 47%), followed by the right coronary (7/17, 41%) and circumflex artery (2/17, 12%). The median dose length product for LE CT scans was 102.5 mGy*cm (IQR, 82.5–115.3 mGy*cm) in the acute phase and 96.6 mGy*cm (IQR, 82.5–106.3 mGy*cm) at follow-up.

**TABLE 1:** Patient Demographics

Patient Parameters	Total (N = 17)
Age (year)	44 (IQR, 36–52)
Sex	
Male	6 (35.3%)
Female	11 (64.7%)
Height (cm)	168 (IQR, 163–171)
Weight (kg)	76.5 (IQR, 66.5–94)
Body mass index (kg/m^2^)	26.8 (IQR, 23.1–30.2)
Obesity (body mass index >30 kg/m^2^)	2 (11.8%)
Arterial hypertension	6 (35.3%)
Dyslipidemia	7 (41.2%)
Diabetes	2 (11.8%)
Systemic inflammatory disease	3 (17.7%)
Smoking history	7 (41.2%)
Family history for coronary artery disease	8 (47.1%)
SCAD type	
1	3 (17.6%)
2	6 (35.3%)
3	5 (29.4%)
4	3 (17.6%)
Affected coronary artery	
Left anterior descending artery	8 (47.1%)
Right coronary artery	7 (41.2%)
Circumflex artery	2 (11.8%)
Concomitant coronary artery disease	1 (5.9%)
Ejection fraction (%)*	55 (IQR, 50–60)
Troponin T (ng/L)†	68 (IQR, 24–392)
Hematocrit during acute phase CT (L/L)	0.40 (IQR, 0.37–0.43)
Hematocrit at follow-up CT (L/L)	0.41 (IQR, 0..40–0.43)
**LE CT scan parameters**	**Total (n = 24)**
Tube current time product (mAs)	48 (IQR, 38–55)
CTDI_vol_ (mGy)	7.6 (IQR, 5.9–8.6)
DLP (mGy*cm)	101.0 (IQR, 79.7–120.0)
SSDE (mGy)	10.4 (IQR, 8.9–11.4)
Signal-to-noise ratio in late enhancement CT	4.5 (IQR, 3.6–6.3)

Values are median and IQR or frequencies and percentages. *Determined by cardiac MRI or echocardiography. †Measured at first clinical presentation after symptom onset. CTDI_vol_, volume CT dose index; DLP, dose length product; IQR, interquartile range; LE, late enhancement; SCAD, spontaneous coronary artery dissection; SSDE, size-specific dose estimate.

### Cardiac MRI Assessment

The median time between symptom onset and MRI in the acute phase was 3 days (IQR, 3–7 days). There was no significant difference between the median number of pathologic segments in acute phase LGE MRI (median, 6 [IQR: 4–8] segments) and follow-up LGE MRI (median, 5 [IQR: 3–7] segments) (*P* = 0.584). In acute phase MRI, MVO was observed in 2 patients (2/16, 12.5%) and edema was present in 14 patients (14/16, 87.5%). At follow-up cardiac MRI, MVO had resolved in all patients, while residual edema was found in 2 patients. There was a significant difference in the median number of segments with edema between acute phase MRI (median, 3 segments [IQR, 2–4 segments]) and follow-up MRI (median, 0 segments [IQR, 0–1 segments]) (*P* = 0.022 (Table 2). In 11 patients with edema in the acute phase (11/14, 78.6%), myocardial segments with edema corresponded to those with LGE. In 2 patients (2/11, 18.2%), edema extended beyond segments with LGE (Table 3), similar to the patients in whom residual edema was found at follow-up (Table 4). Detailed cardiac MRI findings can be found in supplementary table 1, http://links.lww.com/RLI/B32.

**TABLE 2:** Number of Pathologic Myocardial Segments in Acute Phase and Follow-up CT and Cardiac MRI

Number of Pathologic Segments	Median (IQR)	*P* Value
Acute phase CT	8 (IQR, 6–13)	0.104
Follow-up CT	7 (IQR, 4–9)	
Acute phase cardiac MRI (LGE)	6 (IQR, 5–10)	0.584
Follow-up cardiac MRI (LGE)	6 (IQR, 4–8)	
Acute phase cardiac MRI (edema)	3 (IQR, 2–4)	0.022
Follow-up cardiac MRI (edema)	0 (IQR, 0–1)	
Aggregate: acute phase and follow-up CT	8 (IQR, 6–11)	0.014
Aggregate: acute phase and follow-up cardiac MRI (LGE)	6 (IQR, 5–10)	

Values are medians and interquartile ranges (IQR). For LE CT and LGE cardiac MRI, both subepi- and subendocardial myocardial segments were combined. For segments with edema in cardiac MRI, there was no distinction between subepi- and subendocardial. *P* values were calculated using the Wilcoxon signed-rank test. CT, computed tomography; IQR, interquartile range; LGE, late gadolinium enhancement; MRI, magnetic resonance imaging.

**TABLE 3:** Pathologic Myocardial Segments in Acute Phase CT and Cardiac MRI in Patients With Spontaneous Coronary Artery Dissection

	LE CT	LGE Cardiac MRI	Concordance*	Edema in Cardiac MRI	
Participant No.	Pathologic Segments (n = 32 per Patient)		Total/Partial/No (Subepi−/Endocardium or Both)	Segments (n = 16 per Patient)	Overlapping Segments with LGE†
1	13	12	Partial (subepicardium)	4	Yes
2	8	6	Partial (both)	4	No (+1 edema segment)
3	19	6	Partial (both)	10	No (+7 edema segments)
4	13	10	Partial (subepicardium)	6	Yes
5	1	1	Total	1	Yes
6	7	5	Partial (both)	4	Yes
7	13	13	Total	0	-
9	12	12	Total	2	Yes
10	10	10	Total	5	Yes
11	16	2	Partial (both)	2	No (+1 edema segment)
12	7	7	Total	2	Yes
13	2	2	Total	1	Yes
14	9	9	Total	4	Yes
15	6	6	Total	3	Yes
16	3	3	Total	0	-
17	6	6	Total	3	Yes

Values are counts unless otherwise specified. Participant 8 did not receive acute phase imaging. *Intermodality concordance of CT with late gadolinium enhancement cardiac MRI and location of discordance (subepicardium, subendocardium or both). †Overlapping or additional myocardial segments with edema compared to late gadolinium enhancement cardiac MRI. CT, computed tomography; LE, late enhancement; LGE, late gadolinium enhancement; MRI, magnetic resonance imaging.

**TABLE 4:** Pathologic Myocardial Segments in Follow-up CT and Cardiac MRI in Patients With Spontaneous Coronary Artery Dissection

	LE CT	LGE Cardiac MRI	Concordance*	Edema in Cardiac MRI	
Participant No.	Pathologic Segments (n = 32 per Patient)		Total/Partial/No (Subepi-/Endocardium or Both)	Segments (n = 16 per Patient)	Overlapping Segments With LGE†
1	7	6	Partial (both)	2	No (+1 edema segment)
3	8	6	Partial (both)	1	No (+1 edema segment)
8	10	10	Total	0	-
11	5	5	Total	0	-
12	7	7	Total	0	-
13	2	2	Total	0	-
14	10	10	Total	0	-
15	2	2	Total	0	-

Values are counts unless otherwise specified. Participant 8 did not receive acute phase imaging. *Intermodality concordance of CT with late gadolinium enhancement cardiac MRI and location of disconcordance (subepicardium, subendocardium or both). †Overlapping or additional myocardial segments with edema compared to late gadolinium enhancement cardiac MRI. CT, computed tomography; LE, late enhancement; LGE, late gadolinium enhancement; MRI, magnetic resonance imaging.

### CT Image Assessment

#### Acute Phase CT

The median time from symptom onset to CT was 6 days (IQR, 4–8 days). CT identified 14 patients (14/16, 87.5%) with myocardial injury involving 3 or more segments (see Table 3).

#### Follow-up CT

The median time from symptom onset to follow-up CT was 120 days (IQR, 118–123 days). All patients who underwent follow-up CT showed persisting myocardial injury. There was a trend toward smaller injury sizes in follow-up CT, with more pathologic segments in acute phase (median, 8 segments [IQR, 6–13 segments]) than in follow-up CT (median, 7 segments [IQR, 4–9 segments]), but this difference did not reach statistical significance (*P* = 0.104) (see Tables 2, 4). Intrarater and interrater agreement for the assessment of pathologic CT segments was almost perfect with Cohen's κ of 0.982 and 0.970, respectively. Quantitative ECV values can be found in Supplementary Table 2, http://links.lww.com/RLI/B32.

### Comparison Between CT and MRI

#### Acute Phase

The median time between CT and MRI in the acute phase was 3 days (IQR, 2–6 days). Agreement between LE CT and LGE MRI was strong (Cohen's κ = 0.832). Agreement improved to almost perfect when comparing CT to MRI including both LGE and edema (Cohen's κ = 0.944). CT identified all pathologic segments detected by MRI. Total concordance was found in 11 patients (11/16, 68.8%), while partial concordance was found in 6 (6/16, 37.5%). Partial concordance was observed in patients with both LGE and edema, where CT identified more segments with LE than MRI (see Table 3). In 2 patients with MVO (2/16, 12.5%), LE CT images showed a central hypodensity within an area of hyperenhancement, with similar findings in the polar maps. In both patients with MVO, atlas maps did not indicate pathological segments. In another 2 patients (2/16, 12.5%), pathologic MRI segments were identified by LE CT, overlay images, and polar maps but not by atlas maps.

#### Follow-up

The median time between follow-up CT and MRI was 0 days (IQR, 0–1 days). Agreement between LE CT and LGE MRI was almost perfect (Cohen's κ = 0.956) and improved when comparing LE CT with MRI combining both LGE and edema (Cohen's κ > 0.999). In patients without edema and MVO at follow-up (6/8, 75.0%), there was total concordance of CT with MRI. In 2 patients with persistent edema at follow-up (2/8, 25.0%), agreement improved but concordance remained partial (see Table 4).

Acute phase and follow-up CT identified a median of 8 pathologic myocardial segments (IQR, 6–11 segments), which was significantly higher than the number of pathologic myocardial segments detected by acute phase and follow-up LGE MRI (median, 6 segments [IQR, 5–10 segments]) (*P* = 0.014) (see Table 2).

**FIGURE 2:**
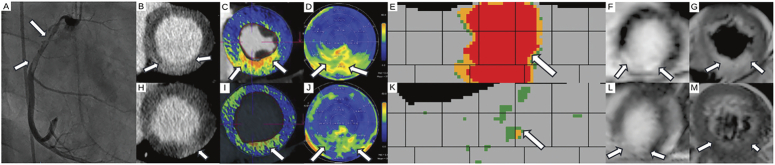
Myocardial injury in spontaneous coronary artery dissection in a 26-year-old male (*patient 11*) with (A) dissection of the proximal and mid right coronary artery in invasive coronary angiography (arrows). B, Late enhancement CT performed 2 days after symptom onset revealed an inferoseptal infarct with elevations of extracellular volume in (C) overlay images, showing voxel-wise color-coded ECV distribution across the myocardium, on (D) polar maps, displaying ECV values per AHA segment in a circular layout, and on (E) atlas maps, which apply 3D segmentation and median filtering to display ECV in a linear 34-layer × 72-sector matrix for enhanced noise suppression and visual clarity (arrows). F, Late gadolinium enhancement cardiac MRI performed 4 days after CT confirmed the inferoseptal infarct showing also edema on T2-weighted images (G) (arrows). Follow-up CT 125 days after symptom onset showed a residual inferior infarct in late enhancement CT (H) in overlay images (I), polar (J) and (K) atlas maps (arrows). (L) Late gadolinium enhancement (arrows) confirms the smaller infarct (M) with no residual edema on T2-weighted cardiac MRI. Retroperitoneal bleeding immediately prior to acute phase CT resulted in a low hematocrit of 0.32 L/L, which normalized to 0.45 during follow-up. The change in hematocrit may have contributed to the differences in extracellular volume elevations between initial and follow-up imaging. The larger infarct size in CT as compared to cardiac MRI can be explained by edema in the acute phase.

**FIGURE 3:**
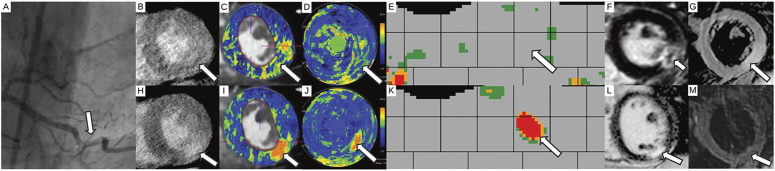
Myocardial injury with microvascular obstruction (MVO) in spontaneous coronary artery dissection in a 45-year-old female (*patient 13*) with (A) dissection of the distal right coronary artery in invasive coronary angiography (arrow). B, Late enhancement CT performed 6 days after symptom onset revealed focal inferolateral hypodensity (arrow) with surrounding late enhancement. Areas of elevated ECV surrounding the central hypodensity are visible in (C) overlay images and on (D) polar maps, with AHA segmental ECV distribution in a circular display (arrows), but no substantial increases are seen in the corresponding atlas map (E), due to smoothing effects from median filtration suppressing small high-contrast areas such as MVO (arrow). F, Late gadolinium enhancement cardiac MRI 2 days prior to CT showed an inferolateral infarct including MVO and edema on (G) T2-weighted cardiac MRI (arrows). In the acute phase, MVO led to diminished perfusion resulting in reduced late enhancement and extracellular volume elevations at the center of the infarct. Nonetheless, the surrounding pathologic myocardium was identifiable via overlay images and polar maps. H, Follow-up CT 117 days after symptom onset revealed an inferolateral myocardial infarct with ECV elevations in (I) overlay images, on (J) polar and on (K) atlas maps (arrows). L, Late gadolinium enhancement (arrows) shows persistent late gadolinium enhancement in the inferolateral wall and (M) T2-weighted cardiac MRI demonstrated resolution of edema (arrows).

**FIGURE 4:**
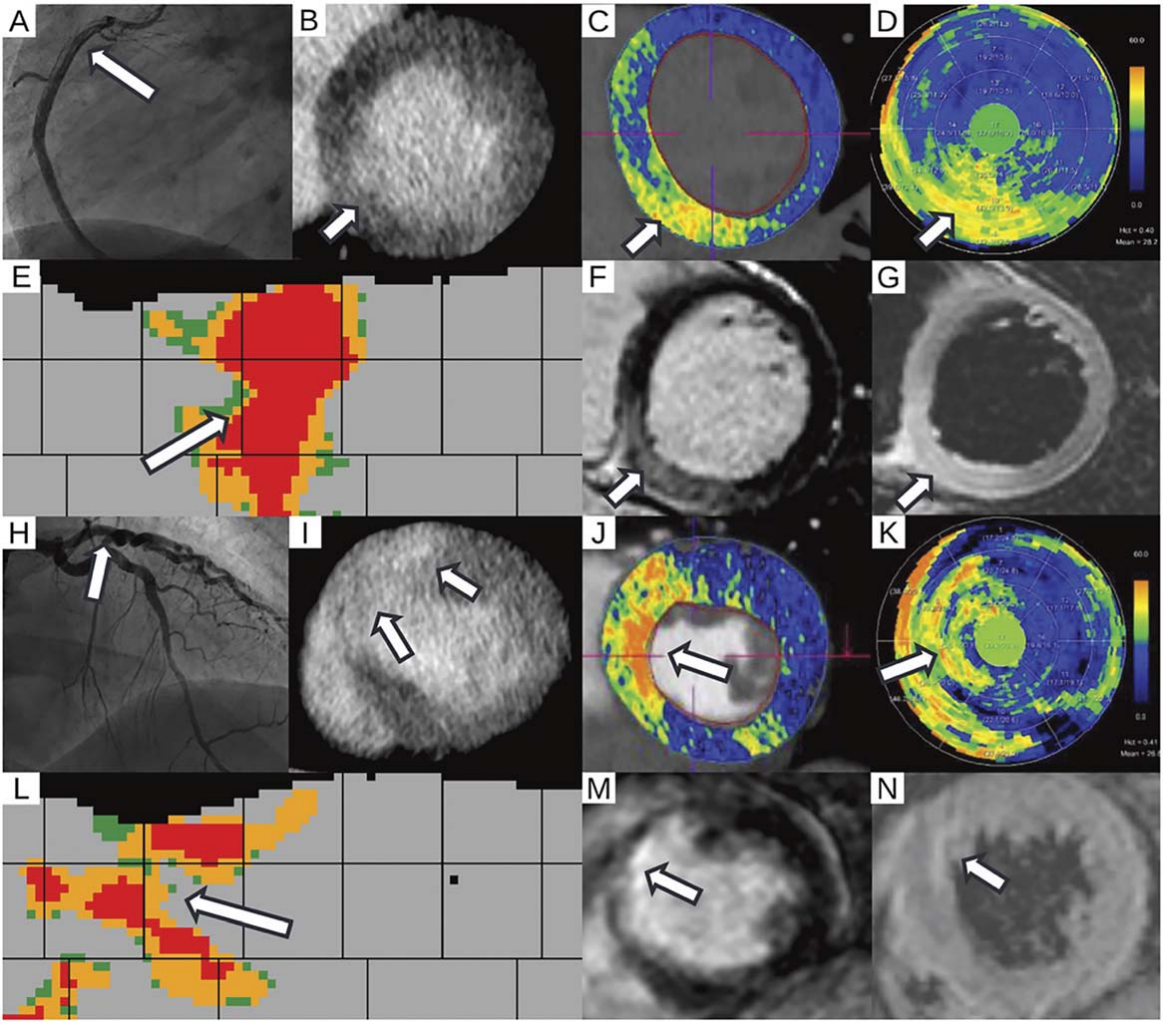
Myocardial injury with and without edema in spontaneous coronary artery dissection. A–G, A 62-year-old female (*patient 2*) with (A) dissection of the proximal right coronary artery in invasive coronary angiography (arrow). (B) Acute phase late enhancement CT performed 3 days after symptom onset revealed a large inferoseptal infarct, also seen in (C) overlay images, (D) polar, and on (E) atlas maps highlighting ECV distribution with noise-suppressed segmental visualization (arrows). F, Late gadolinium enhancement cardiac MRI performed on the same day as acute phase LE CT confirmed a smaller inferoseptal infarct with edema on (G) T2-weighted cardiac MRI (arrows), indicating partial concordance between CT and cardiac MRI. The larger infarct size in CT could be explained by edema in the acute phase. H–N, A 53-year-old female (*patient 8*) with (H) dissection of the mid left anterior descending artery in invasive coronary angiography (arrow). I, Follow-up CT 116 days after symptom onset revealed an anteroseptal infarct also seen in (J) overlay images, on (K) polar, and on (L) atlas maps depicting filtered segmental ECV elevations. M, Late gadolinium enhancement cardiac MRI performed one the same day as follow-up LE CT depicted the corresponding infarct without edema on (N) T2-weighted cardiac MRI (arrows), indicating total concordance between CT and cardiac MRI.

Detailed results of intermodality assessment are provided in Supplementary Table 3, http://links.lww.com/RLI/B32. Representative image examples are depicted in Figures 2-4.

## DISCUSSION

This prospective single-center study is the first to evaluate the accuracy of LE CT with PCD-CT for diagnosing myocardial injury in a specific patient population and introduces a new way of visualization of the ECV distribution throughout the myocardium (*atlas maps*). In patients with SCAD, we found strong overall agreement between LE PCD-CT and cardiac MRI for detecting acute phase myocardial injury, which increased to almost perfect during follow-up. Comparing LE CT with cardiac MRI combining both LGE and edema improved agreement between modalities both acute and during follow-up. Atlas maps were helpful for an easy and more intuitive depiction of pathological myocardial segments with LE CT.

LE imaging can increase the diagnostic yield of CT in patients with acute chest pain.^15,16^ Palmisano et al^15^ showed that in patients with acute chest pain and mild troponin elevation but without obstructive coronary artery disease, LE CT enabled the diagnosis of myocarditis in 22, takotsubo cardiomyopathy in 4, amyloidosis in 3, myocardial infarction with nonobstructed coronary arteries in 3, and dilated cardiomyopathy in 2 patients. Our study expands the concept of Palmisano et al^15^ to patients with SCAD. In clinical practice, LE CT could be added to the protocol if coronary CT angiography is negative, indeterminate, or nondiagnostic. LE CT can also serve as an alternative when cardiac MRI is unavailable or contraindicated. It can be performed with minimal additional time (5 minutes after contrast media administration) and moderate additional radiation exposure (DLP 101 mGy*cm). These findings underscore the potential of LE CT as a rapid and accessible imaging option in the acute phase of SCAD, particularly in emergency settings where MRI may not be immediately available and broadens the clinical utility of cardiac CT by enabling myocardial tissue characterization in acute care scenarios.

We introduced atlas maps as a novel method for a fast and intuitive visualization of myocardial injury with CT. Like polar maps, atlas maps offer a 2-dimensional representation of ECV alterations across the segmentally divided myocardium. However, they incorporate additional filtration to reduce noise and display ECV elevations in a simplified, color-coded format using 3 distinct thresholds. By arranging myocardial segments in horizontal rows rather than a circular layout, atlas maps aim to streamline visual interpretation. They may provide improved precision through higher-resolution 3D segmentation and more effective noise reduction. Nevertheless, 1 observed limitation was that excessive filtering may obscure small pathologic segments. As such, atlas maps should not be interpreted in isolation but rather as part of a comprehensive image assessment that includes LE images and overlays. While they offer a standardized and visually simplified display of ECV abnormalities—useful for supporting interpretation and follow-up—they are designed to complement, not replace, source imaging datasets.

We found differences in assigning pathologic myocardial segments between LE CT images and polar and atlas maps. In 1 patient, LE CT images appeared normal but polar and atlas maps correctly identified pathologic myocardial segments. Conversely, in 2 patients, atlas and polar maps showed no abnormalities but LE CT images revealed myocardial hypodensities. One patient suffered from large retroperitoneal bleeding immediately prior to CT and hence, with a low hematocrit. Because ECV calculation is based on actual hematocrit levels, atlas maps and polar maps may have overestimated the true infarct size in this patient. The additional distribution volume available for contrast media and coronary vasodilation in individuals with low hematocrit levels due to bleeding increases myocardial perfusion of contrast media.^28^

Edema on cardiac MRI was present in most patients in the acute phase, leading to discrepancies in comparisons of LE CT with LGE MRI, while agreement improved when edema subsided at follow-up. Extracellular contrast media distributes more readily in pathologic myocardium due to cell membrane rupture and interstitial expansion, with delayed washout from necrotic tissue causing hyperenhancement.^11^ In some patients, edema may have led to overestimation of infarct size due to diffuse expansion of the extravascular extracellular space, a phenomenon known from cardiac MRI.^29,30^ Importantly, LE CT was acquired 5 minutes after contrast administration, while LGE MRI was performed 10 minutes after injection. This temporal difference may result in LE CT capturing early-phase extracellular contrast distribution, including reversible changes such as edema, while LGE MRI reflects predominantly irreversible injury. This timing difference could partially explain why LE CT occasionally detected additional enhancement not visualized on LGE MRI. Notably, agreement improved when edema was considered alongside LGE on cardiac MRI, supporting the interpretation that LE CT—with the contrast media protocol used herein—visualizes both scar and edema in the acute setting.

LE CT identified 2 patients with MVO in the acute phase, confirmed with cardiac MRI. This finding was evident in LE CT images and in polar maps, in line with previous CT studies^1^ and comparable to gadolinium-enhanced MRI,^2^ but was not seen on atlas maps. The failure of atlas maps to depict MVO was likely due to the median filtering process, which removed the narrow ring-like area of elevated ECV surrounding a central hypodense core. This highlights a potential limitation of the filtering technique used in atlas maps, which, while effective in reducing noise, may suppress diagnostically relevant features. atlas maps should not be used and interpreted in isolation.

We recognize several study limitations. First, this was a single-center investigation using a PCD-CT scanner from 1 vendor, which may limit generalizability. Second, although the number of included patients was relatively small compared to studies on more prevalent myocardial disease,^25,26^ this must be viewed in light of the low prevalence of SCAD.^1,2^ Given the rarity of the disease, we believe that including 17 patients—many with both CT and MRI in both acute and follow-up phases—represents a considerable cohort. No formal power calculation was performed because of the exploratory nature of the study and lack of prior data; however, the intraindividual design and comprehensive imaging protocol add to its strength. Third, several patients were lost to follow-up or could not undergo cardiac MRI, which may reflect the psychologically challenging situation of patients with SCAD, as previously described.^1^ Fourth, cardiac MRI–derived ECV was only available in a subset of patients, which precluded direct quantitative comparison with CT-derived ECV, as recently performed in other contexts.^25^ Consequently, only qualitative assessments of LE CT including polar and atlas maps could be performed. Additionally, thresholds for pathological CT-derived ECV were chosen conservatively based on literature and technical considerations but remain nonstandardized and require validation in larger multicenter studies. Finally, atlas maps were generated using proprietary algorithms that are not yet commercially available.

In conclusion, this study demonstrated strong agreement of LE imaging with PCD-CT compared to cardiac MRI in detecting myocardial injury in patients with SCAD. Polar and newly introduced atlas ECV maps were helpful in the identification of pathologic myocardium, the latter helping in streamlining diagnostic interpretation of ECV maps in an intuitive way. Larger multicenter studies are needed to further validate the potential of LE imaging with CT in this population.

## ORCID ID

Hatem Alkadhi https://orcid.org/0000-0002-2581-2166

## Supplementary Material

SUPPLEMENTARY MATERIAL
