# Supplementary material for: Myocardial Late Enhancement With Photon-Counting Detector CT in Spontaneous Coronary Artery Dissection: Prospective Comparison With Cardiac MRI
Source: Invest Radiol. 2025 Apr 25;61(1):32–40. doi: 10.1097/RLI.0000000000001203 (PMC12662126; doi:10.1097/RLI.0000000000001203)
Supplement: SUPPLEMENTARY MATERIAL [file rli-61-32-s001.docx]

**Supplementary material**

**
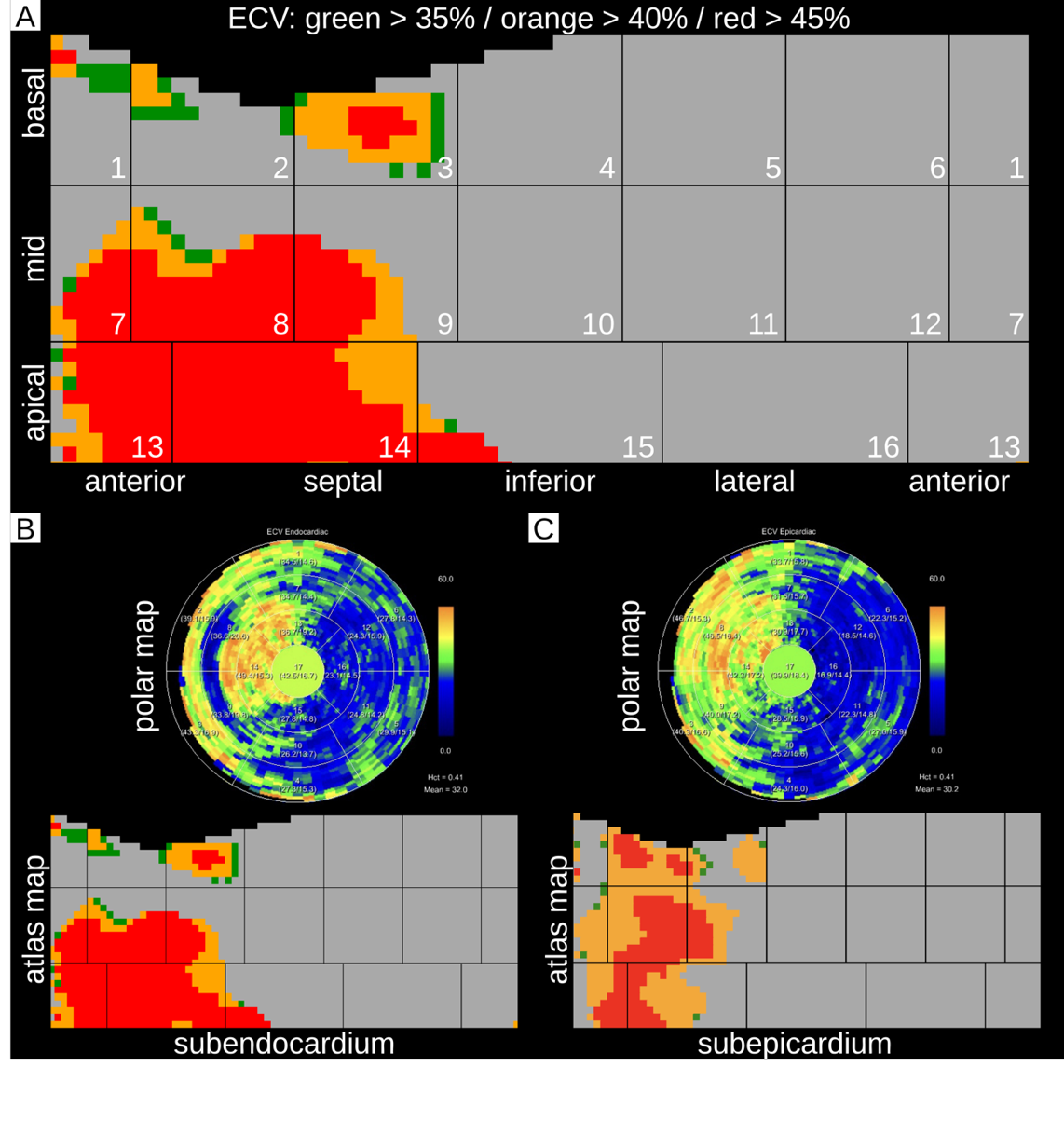
**

**Supplementary figure:** (A) Atlas maps are two-dimensional visual representations of myocardial extracellular volume (ECV) elevations. Similar to polar maps, segments are numbered according to the American Heart Association in ascending order from base to apex and from anterior, septal, inferior, to lateral. The myocardium is depicted as a rectangular plane cut open at segments 1, 7, and 13. Segments are delineated as either square regions (at the base and midventricular level) or rectangular regions (at the apical level), with white numbers indicating segment numbers. ECV elevations (in percentage) are color-coded: red indicates values >45%, orange >40%, and green >35%. The black stripe at the top of the map denotes the heart base. (B-C) Bottom row: Polar and atlas maps are generated for the (B) subendocardium (10-50% of the myocardial wall thickness) and (C) subepicardium (50-90% of the myocardial wall thickness), excluding the inner- and outermost 10% to avoid inclusion of artifacts that might interfere with ECV calculations.

**Supplementary table 1:** Edema and microvascular obstruction in acute phase and follow-up MRI.

| **Patient** | **MVO**  Acute phase | **Edema**  Acute phase | **^1^Segments**  Acute phase | | **MVO**  Follow-up | | **Edema**  Follow-up | | **^1^Segments**  Follow-up |
| --- | --- | --- | --- | --- | --- | --- | --- | --- | --- |
| 1 | yes | yes | 5;6;11;15 | no | | yes | | 11;15 | |
| 2 | no | yes | 4;9;10;15 | - | | - | | - | |
| 3 | no | yes | 3;4;8-15 | no | | yes | | 8 | |
| 4 | no | yes | 7-9;13-15 | - | | - | | - | |
| 5 | no | yes | 15 | - | | - | | - | |
| 6 | no | yes | 3;4;9;10 | - | | - | | - | |
| 7 | no | no | n | - | | - | | - | |
| 8 | - | - | - | no | | no | | - | |
| 9 | no | yes | 11;16 | - | | - | | - | |
| 10 | no | yes | 2-4;8;9 | - | | - | | - | |
| 11 | no | yes | 14;15 | no | | no | | - | |
| 12 | no | yes | 10;15 | no | | no | | - | |
| 13 | yes | yes | 11 | no | | no | | - | |
| 14 | no | yes | 4;5;15;16 | no | | no | | - | |
| 15 | no | yes | 11;15;16 | no | | no | | - | |
| 16 | no | no | n | - | | - | | - | |
| 17 | no | yes | 1;6;12 | - | | - | | - | |

Note.- “n” denotes no pathologic segments recorded. MVO=microvascular obstruction

^1^American Heart Association myocardial segments with edema

**Supplementary table 2:** Quantitative extracellular volume analysis in acute and follow-up LE CT.

| **Variable** | **Median (IQR)** | **P-value** |
| --- | --- | --- |
| Subendocardial ECV > 45% in acute phase CT [%] | 3.2 (IQR, 2.3, 10.6) | .012 |
| Subepicardial ECV > 45% in acute phase CT [%] | .9 (IQR, 0, 3.9) |  |
| Subendocardial ECV > 40% in acute phase CT [%] | 9.4 (IQR, 4.6, 20.8) | .004 |
| Subepicardial ECV > 40% in acute phase CT [%] | 3.0 (IQR, 1.2, 12.5) |  |
| Subendocardial ECV > 35% in acute phase CT [%] | 16.6 (IQR, 7.8, 22.8) | .007 |
| Subepicardial ECV > 35% in acute phase CT [%] | 6.6 (IQR, 2.8, 14.2) |  |
| Subendocardial median ECV in acute phase CT [%] | 28.1 (IQR, 26.1, 28.9) | .770 |
| Subendocardial median ECV in follow-up phase CT [%] | 29.0 (IQR, 27.4, 31.4) |  |
| Subepicardial median ECV in acute CT phase [%] | 28.1 (IQR, 26.1, 28.9) | .625 |
| Subepicardial median ECV in follow-up CT phase [%] | 29.7 (IQR, 27.5, 31.4) |  |
| Note.- Values are median and interquartile range (IQR)*.* P-values were calculated using the Wilcoxon signed-rank test. ECV= extracellular volume, IQR=interquartile range | | |

**Supplementary table 3:** Detailed description of each patient including acute phase and follow-up CT and MRI findings.

| **Patient** | **Involved coronary segments in invasive angiography (AHA)** | **Time between symptom onset**  **and acute phase CT [days]** | **^1^Time between acute phase CT**  **and MRI [days]** | **Time between follow-up CT**  **and MRI [days]** | **Pathologic myocardial segments**  **in acute phase CT and MRI** | | | | **^4^Concordance of acute phase CT with MRI** | **Comment on acute phase imaging** | **Pathologic myocardial segments**  **in follow-up CT and MRI** | | | | **^4^Concordance of follow-up CT with MRI** | **Comment on follow-up imaging** |
| --- | --- | --- | --- | --- | --- | --- | --- | --- | --- | --- | --- | --- | --- | --- | --- | --- |
|  |  |  |  |  | **^2^Subendocardium -  CT** | **^3^Subendocardium –**  **LGE MRI** | **^2^Subepicardium -  CT** | **^3^Subepicardium -  LGE MRI** |  |  | **^2^Subendocardium -  CT** | **^3^Subendocardium –**  **LGE MRI** | **^2^Subepicardium -  CT** | **^3^Subepicardium -  LGE MRI** |  |  |
| 1 | 6,7 | 6 | -3 | 1 | 4-6;10;11;15;16 | 4-6;10;11;15;16 | 5;6;10;11;15;16 | 5;10;11;15;16 | 2 | partial concordance:  2 pathologic epicardial MRI segments not identified in atlas maps but in LE images and in polar maps,  1 additional pathologic epicardial segment in LE and atlas/polar maps,  MVO and edema in MRI | 4-6;11;15 | 4-6;11 | 4-6;11;15 | 4-6;11 | 2 | partial, improved concordance:  residual edema,  no MVO in follow-up MRI |
| 2 | 1 | 3 | 0 | - | 4;9;10;15 | 4;10;15 | 4;9;10;15 | 4;10;15 | 2 | partial concordance:  1 additional endocardial pathologic segment in LE and atlas/polar maps,  1 additional epicardial pathologic segment in LE and atlas/polar maps,  edema in MRI | - | - | - | - | - | - |
| 3 | 8 | 5 | 2 | 0 | 3;4;8-16 | 13-15 | 3;8-10;13-16 | 13-15 | 2 | partial concordance:  8 additional endocardial pathologic segments in LE and atlas/polar maps,  5 additional epicardial pathologic segments in LE and atlas/polar maps,  edema in MRI | 8;13;14;15 | 13;14;15 | 8;13;14;15 | 13;14;15 | 2 | partial, improved concordance:  residual edema in follow-up MRI |
| 4 | 6,7 | 7 | 0 | - | 7-9;13-16 | 7-9;13-16 | 8;9;13-15 | 13-15 | 2 | partial concordance:  2 additional epicardial pathologic segments in LE and atlas/polar maps,  edema in MRI | - | - | - | - | - | - |
| 5 | 3 | 4 | -3 | - | 15 | 15 | 15 | 15 | 1 | total concordance | - | - | - | - | - | - |
| 6 | 1 | 3 | -1 | - | 3-5;9;10 | 3-5;9;10 | 3;9 | n | 2 | partial concordance:  2 additional epicardial pathologic segments in LE and atlas/polar maps,  edema in MRI | - | - | - | - | - | - |
| 7 | 5 | 11 | 5 | - | 5;8;10;11;13-15 | 5;8;10;11;13-15 | 5;8;11;13-15 | 5;8;11;13-15 | 1 | total concordance:  2 pathologic MRI segments identified in LE and polar but not in atlas map, filtration issue in atlas map ? | - | - | - | - | - | - |
| 8 | 7,8 | 4 | - | 0 | - | - | - | - | - | - | 2;3;8;9;13;14 | 2;3;8;9;13;14 | 3;8;9;14 | 3;8;9;14 | 1 | total concordance |
| 9 | 11 | 4 | -2 | - | 4;10-12;15;16 | 4;10-12;15;16 | 10-12;15;16 | 10-12;15;16 | 1 | total concordance | - | - | - | - | - | - |
| 10 | 7 | 12 | 10 | - | 2-4;8;9 | 2-4;8;9 | 2-4;8;9 | 2-4;8;9 | 1 | total concordance:  4 pathologic MRI segments identified in LE and polar but not in atlas map, filtration issue in atlas map ? | - | - | - | - | - | - |
| 11 | 1,2 | 2 | -4 | 0 | 3-5;9-11;14-16 | 15 | 3;4;9;10;14-16 | 15 | 2 | partial concordance:  7 additional endocardial pathologic segments in atlas/polar maps,  5 additional epicardial pathologic segments in atlas/polar maps,  pathologic segments not identified in LE,  edema in MRI | 4;10;15 | 4;10;15 | 10;15 | 10;15 | 1 | total concordance:  no edema in follow-up MRI |
| 12 | 4 | 1 | 0 | 0 | 10;11;15;16 | 10;11;15;16 | 10;15;16 | 10;15;16 | 1 | total concordance | 10;11;15;16 | 10;11;15;16 | 10;15;16 | 10;15;16 | 1 | total concordance:  no edema in follow-up MRI |
| 13 | 14 | 6 | 2 | 0 | 11 | 11 | 11 | 11 | 1 | total concordance:  1 pathologic segment not identified in atlas maps but in LE images and in polar maps,  MVO and edema in MRI | 11 | 11 | 11 | 11 | 1 | total concordance:  no MVO/no edema in follow-up MRI |
| 14 | 7,8 | 8 | -25 | 0 | 4;5;10;11;15;16 | 4;5;10;11;15;16 | 5;11;15 | 5;11;15 | 1 | total concordance | 4;5;10;11;15;16 | 4;5;10;11;15;16 | 4;5;11;15 | 4;5;11;15 | 1 | total concordance:  no edema in follow-up MRI |
| 15 | 6 | 8 | 5 | 1 | 11;15;16 | 11;15;16 | 11;15;16 | 11;15;16 | 1 | total concordance | 11 | 11 | 11 | 11 | 1 | total concordance:  no edema in follow-up MRI |
| 16 | 15 | 28 | 20 | - | 5;6;12 | 5;6;12 | n | n | 1 | total concordance | - | - | - | - | - | - |
| 17 | 13 | 26 | 22 | - | 1;6;12 | 1;6;12 | 1;6;12 | 1;6;12 | 1 | total concordance | - | - | - | - | - | - |

Note.- “n” denotes no pathologic segments recorded. AHA=American Heart Association, LE= late iodine enhancement, LGE=late gadolinium enhancement

^1^negative values indicate that CT was acquired before MRI

^2^American Heart Association myocardial pathologic segments identified in CT

^3^American Heart Association myocardial segments with LGE identified in MRI

^4^Concordance of LE CT with LGE MRI: 1=total, 2=partial, 3=no concordance
